# Achieving tolerance modifies cancer susceptibility profiles in liver transplant recipients

**DOI:** 10.1002/cam4.5271

**Published:** 2022-10-07

**Authors:** Mamatha Bhat, Elisa Pasini, Preya Patel, Jeffrey Yu, Cristina Baciu, Sunil M. Kurian, Josh Levitsky

**Affiliations:** ^1^ Multi Organ Transplant Program and Division of Gastroenterology University Health Network and University of Toronto Toronto Canada; ^2^ Scripps Clinic Bio‐Repository & Transplantation Research, Scripps Clinic & Green Hospital La Jolla California USA; ^3^ Division of Gastroenterology & Hepatology, Department of Medicine Northwestern University Feinberg School of Medicine Chicago Illinois USA; ^4^ Comprehensive Transplant Center, Department of Surgery Northwestern University Feinberg School of Medicine Chicago Illinois USA

## Abstract

Long‐term survival of transplant recipients is significantly impacted by malignancy. We aimed to determine whether calcineurin inhibitor (CNI)‐treated recipients converted to and weaned off molecular target of rapamycin inhibitor (mTOR‐I) therapy have favorable changes in their molecular profiles in regard to malignancy risk. We performed gene expression profiling from liver biopsy and blood (PBMC) specimens followed by network analysis of key dysregulated genes, associated diseases and disorders, molecular and cellular functions using IPA software. Twenty non‐immune, non‐viremic patients were included, and 8 of them achieved tolerance. Two comparisons were performed: (1) tolerance time point vs tacrolimus monotherapy and (2) tolerance time point vs sirolimus monotherapy. Upon achieving tolerance, IPA predicted significant activation of DNA damage response (*p* = 5.40e‐04) and inhibition of DNA replication (*p* = 7.56e‐03). Conversion from sirolimus to tolerance showed decrease in HCC (*p* = 1.30e‐02), hepatic steatosis (*p* = 5.60e‐02) and liver fibrosis (*p* = 2.91e‐02) associated genes. In conclusion, this longitudinal study of patients eventually achieving tolerance reveals an evolving molecular profile associated with decreased cancer risk and improved hepatic steatosis and liver fibrosis. This provides a biological rationale for attempting conversion to mTOR‐I therapy and tolerance following liver transplantation particularly in patients at higher risk of cancer incidence and progression post‐transplant.

## Graphical abstract

Achieving tolerance modifies cancer susceptibility profiles in liver transplant recipients. Liver transplant patients who weaned off sirolimus therapy have favorable changes in their molecular profiles in regard to malignancy risk.

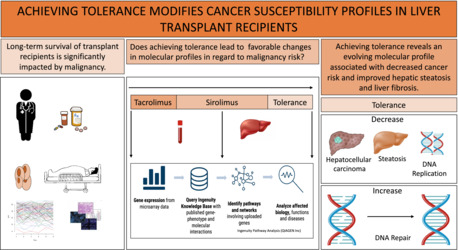

## 1 INTRODUCTION

De novo malignancies arise twice as often in liver transplant (LT) recipients when compared with the general population, significantly impacting long‐term transplant survival.^1^, ^2^, ^3^ In fact, LT recipients have a 10‐year incidence rate of 11.5% versus a 6.5% cancer incidence in the general population after the age 50.^1^ A diagnosis of malignancy is associated with 25% mortality in the first year, and as high as 57.6% at 5 years after a post‐transplant cancer diagnosis.^1^ Therefore, a cancer diagnosis is associated with a significantly greater risk of adverse outcome, with a 2.84 times higher risk of cancer‐related death in LT patients than the general population.^4^

Though several factors have been shown to contribute to the development of malignancies, immunosuppression is a key risk factor.^5^ A higher degree of immunosuppression is associated with an increased risk of malignancy across solid organ transplant recipients. Overall, the decreased cancer‐sensing function of the immune system in immunosuppressed transplant recipients necessitates screening for specific types of cancer more commonly than in the general population. Additionally, the often‐aggressive nature of cancers diagnosed in transplant recipients results in higher mortality.^6^ For example, recurrent hepatocellular carcinoma (HCC) is typically more aggressive that pre‐transplant HCC likely due to immunosuppression.

Given this increased susceptibility to cancer, transplant specialists attempt to minimize immunosuppression in LT recipients, as long as there is low concern for developing rejection. The mammalian target of rapamycin inhibitors (mTORi) are immunosuppressive agents with evidence of concomitant antineoplastic effect, thereby potentially serving a dual role in recipients.^7^ While controversial and not clinically proven effective, patients at higher risk of de novo or recurrent malignancy are often converted from a CNI to an mTORi for a potential chemopreventive effect. While complete withdrawal of immunosuppression is ideal, it is feasible only in select LT recipients.^8^ Nonetheless, the achievement of tolerance in LT recipients is a natural experiment that provides the opportunity to understand the mechanistic basis of cancer susceptibility and metabolic disease in transplant recipients.

Therefore, the aim of our exploratory study was to determine whether patients converted from putatively pro‐neoplastic (CNI) to anti‐neoplastic (mTORi) IS therapy and then weaned off immunosuppression have favorable changes in their molecular profiles in regard to malignancy risk, using a Network analysis approach.

## 2 MATERIALS AND METHODS

### 2.1 Clinical protocol

This cohort included LT recipients enrolled in previously published clinical trials.^9^, ^10^ Twenty LT recipients, were converted from tacrolimus monotherapy to sirolimus for the indication of tacrolimus toxicity, with clinical characteristics as previously described,^9^ Briefly, for conversion to sirolimus, a dose of 2 or 3 mg (< or ≥100 kg body weight)/day was initiated with weekly sirolimus trough level monitoring. When patients reached ≥5 ng/ml, tacrolimus was discontinued followed by initial weekly laboratory tests to monitor sirolimus trough levels (goal 5–8 ng/ml) for one month, then monthly monitoring. Liver/renal function tests, lipid levels, urine protein: creatinine ratios and any signs of sirolimus toxicities were recorded. A subset of these twenty LT recipients^8^ were successfully weaned off of sirolimus.^10^ For all the patients enrolled, baseline physical examination and laboratory tests were performed and if acceptable, a baseline liver biopsy was performed within one month. Sirolimus was slowly reduced over a period of time of approximately 3–6 months as per our previously described protocol. If liver tests were normal, sirolimus was discontinued completely. Subjects were seen six months later and all baseline assessments were repeated for the final study visit 12 months post‐full withdrawal. Liver tests were performed every 2 weeks at the patient's local laboratory throughout the trial. At any concern for rejection, defined by abnormal liver tests, liver biopsy and blood/tissue biomarkers assays were performed. If rejection was diagnosed, the patient was withdrawn from the study. The study inclusion and exclusion criteria for both studies were as previously described.^9^, ^10^ A written informed consent was obtained from patients for use of liver biopsy tissue and peripheral blood samples. The study protocol was approved by the Northwestern Institutional Review Board and was performed in accordance with the Declaration of Helsinki (https://clinicaltrials.gov/ct2/show/NCT02062944).

### 2.2 Gene expression microarrays

Liver biopsy tissue and peripheral blood were collected at three time points: (1) prior to conversion from tacrolimus to sirolimus, (2) 6 months following conversion to sirolimus, and (3) 6 months following weaning off of sirolimus.^10^ RNA extraction from blood samples was performed using the PaxGene blood RNA kit. RNA was extracted from snap‐frozen liver tissue and purified using RNeasy Mini Kit (Qiagen). RNA quality was verified by Nanodrop spectrophotometer (VWR) and Bioanalyzer (Agilent). Gene expression profiling was performed with Affymetrix HT HG‐U133 Plus PM arrays (Affymetrix) following standard protocols. Two different time points were compared as follows: (1) tolerance time point vs tacrolimus and (2) tolerance time point vs sirolimus. Gene expression analysis was performed as previously described.^10^

### 2.3 Protein–Protein interaction network analysis and pathway enrichment

The list of differentially expressed genes from the previous analysis^4^ with FDR correction <0.05 and corresponding log_2_(fold change) were used as input for pathway and network analyses with Ingenuity Pathway Analysis (QIAGEN Inc., https://www.qiagenbio‐ informatics.com/products/ingenuity‐pathway‐analysis). The core analysis module in IPA was performed to identify differentially regulated diseases and biological functions, and gene networks illustrating the genes and their interactors based on Fisher's exact test, as previously reported.^11^ The network is then shown as a graph representing the molecular relationships/interactions as an edge (line) between genes or gene products (nodes). The connectivity of these nodes representing the genes is based on the data collected in the IPA knowledge base. The node color indicates an up‐modulation (red) or down‐modulation (green). Edges are displayed with various colors or labels to better describe the nature of the relationship between the nodes. The Molecular Activity Predictor (MAP) tool was used to predict the crosstalk relationship among our genes and their interactors in the different protein–protein interaction networks. The molecular activity prediction was performed on all the networks considered. Each network considered significant for the study was then overlaid with the diseases and functions database to identify the effect of conversion to sirolimus and achievement of tolerance, and the most common relevant process was identified.

The z‐score was obtained as previously reported. A z‐score ≤−2 (inhibition) or *z*‐score ≥2 (activation) is considered strongly significant of a predicted change of status. Outside these values, the prediction of activation or inhibition is still present, but less confident. In some cases, the *z*‐score cannot be calculated, if there is not enough information stored in the IPA knowledge base.

## 3 RESULTS AND DISCUSSION

Twenty non‐immune (without autoimmune liver disease as indication for transplant), non‐viremic patients (without active, ongoing hepatitis B virus (HBV) or hepatitis C virus (HCV) infection) (age 57.2 ± 8; 95% Caucasian; 3.5 ± 2.1 years post‐LT and with no additional immunosuppressive therapy at the moment of enrollment) treated with tacrolimus were successfully converted to sirolimus,^9^ and 8 of them achieved tolerance.^9^, ^10^ No differences were identified between the two groups (achieved tolerance vs not achieved tolerance) with respect to median age 63.7 (47.3–76.3) versus 62.7 (44–67.6), time on sirolimus monotherapy 4.2 (0.62–5.5) versus 4.1 (2.5–5.4) years, or time from LT to weaning. 8.1 (4.5–12.0) versus 6.9 (3.0–10.9), respectively, as described before.^10^

### 3.1 Tolerance versus tacrolimus monotherapy

**FIGURE 1:**
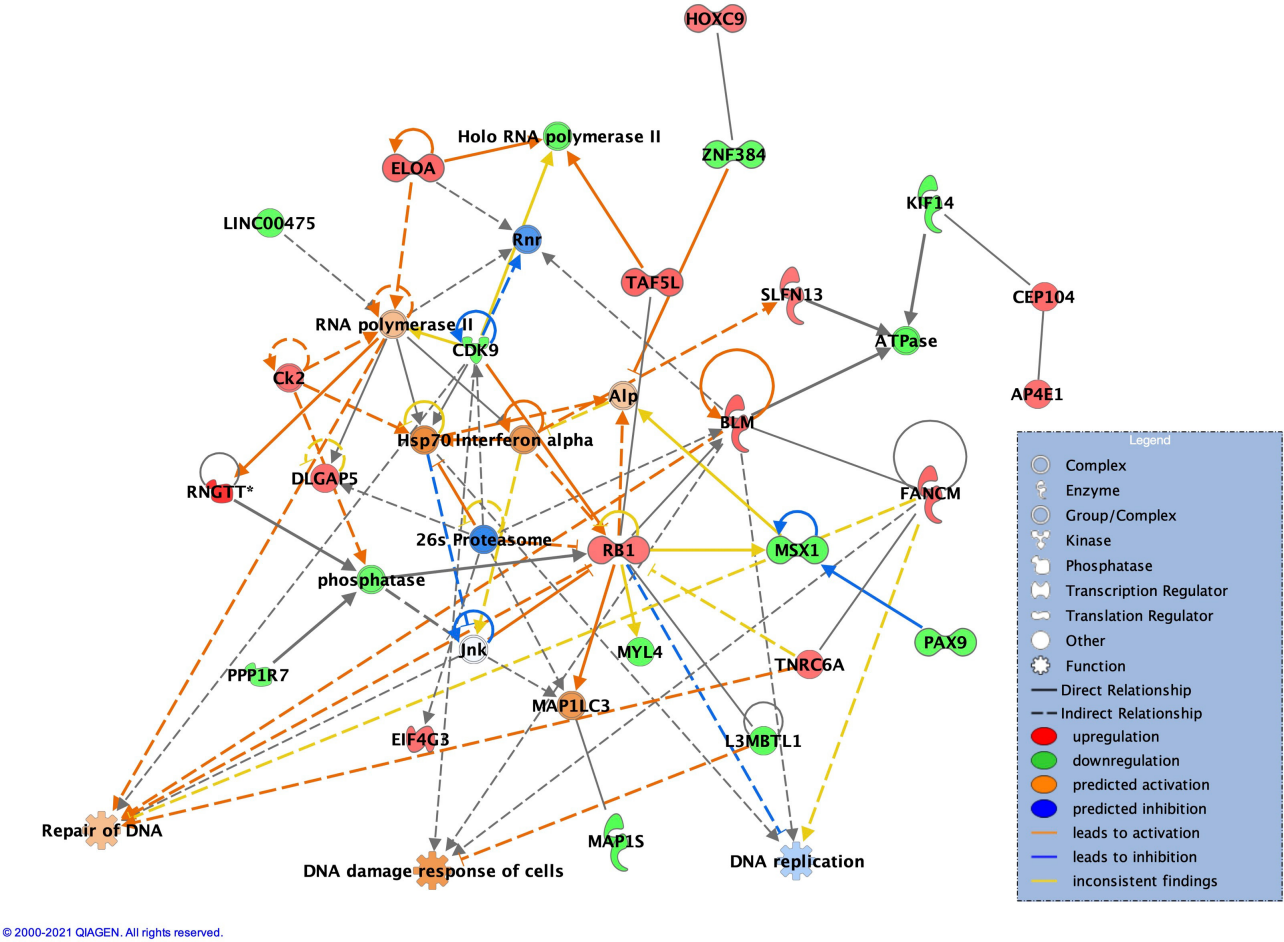
The achievement of tolerance compared with tacrolimus affected genes involved in DNA damage response and DNA replication in the liver tissue. Comparison of tolerance versus tacrolimus timepoint. Tolerance is predicted to improve Repair of DNA (*p* = 2.78E‐04), DNA damage response (*p* = 5.40e‐05) and inhibit DNA replication (*p* = 7.56e‐03) of liver cells based on the overlay with disease and function database. Network 3 in IPA: Cell cycle, DNA replication and Repair

276 genes were found differentially expressed between tolerance versus tacrolimus monotherapy in liver biopsies, with 96 genes being upregulated and 180 downregulated. These were associated with cancer (*p*‐value range = 1.08e‐02—1.60e‐13), gastrointestinal disease (p‐value range = 1.08e‐02—1.30e‐11) as top diseases, and with cell cycle (*p*‐value range = 1.08e‐02— 2.57e‐04), DNA replication, recombination and repair (*p*‐value range = 1.08e‐02—2.57e‐04) as top molecular and cellular functions. Network analysis identified DNA Replication, Recombination, and Repair, Cell Cycle as one of the most significant networks (Figure 1) associated with the DEGs (score = 40), with several pro‐cancer and anti‐cancer genes being involved (Appendix S1).

**FIGURE 2:**
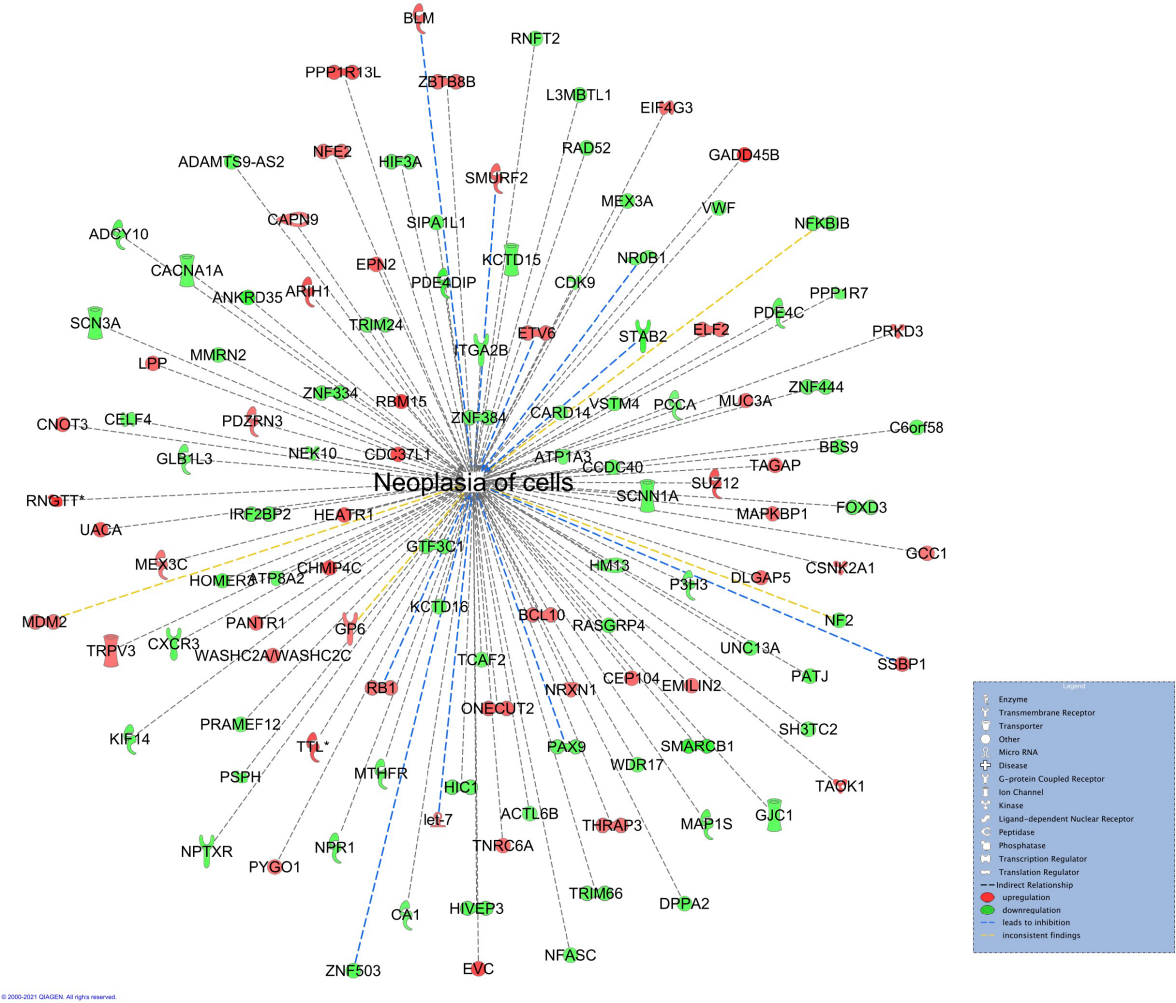
Tolerance is predicted to inhibit Neoplasia of cells (*p* = 3.15e‐04) in liver tissue. Comparison of tolerance versus tacrolimus timepoint

When overlaying this network with diseases and functions, repair of DNA was predicted to be activated (*p* = 2.78e‐04) due to the upregulation of transcription regulators RB1 (RB transcriptional Corepressor 1, FC = 2.20) and ELOA (Elongin A, FC = 2.41), and to increased expression of TNRC6A (Trinucleotide Repeat Containing Adaptor 6A, FC = 2.26). The downregulated L3MBTL1 (L3MBTL Histone Methyl‐Lysine Binding Protein 1, FC = 0.43) gene was linked to enriched DNA damage response (*p* = 5.40e‐04) that was predicted to be highly activated. Upregulated RB1 and SLFN13 (FC = 2.20) were associated with DNA replication, predicted to be enriched (*p* = 7.56e‐03) and inhibited. Our analysis also revealed neoplasia of cells, as a process, being enriched (*p* = 3.15e‐04) and highly inhibited (*z‐*score = −2.1) by DEGs involved (Figure 2). Of the 121 DEGs related to neoplasia (49 upregulated, 72 downregulated), 10 genes were predicted by IPA to significantly reduce this process (Appendix S1).

Similarly, when using PBMC samples, we found tolerance association with cancer and gastrointestinal diseases being significant, with p‐values ranging from 4.21e‐03 to 1.82e‐20 and from 3.20e‐03 to 4.60e‐6, respectively. As in liver tissue, network analysis identified DNA replication, Recombination and Repair, Cell morphology, Cellular Assembly and Organization as one of the top networks corresponding to the DEGs between tolerance and tacrolimus in blood samples (Appendix S1). By employing IPA overlapping tool with diseases and functions, a significant activation of DNA damage response of cells (*p* = 2.92e‐06), repair of DNA (*p* = 4.20e‐04), double‐stranded DNA break repair (*p* = 2.93e‐03) were revealed (Figure S1).

### 3.2 Tolerance versus sirolimus monotherapy

**FIGURE 3:**
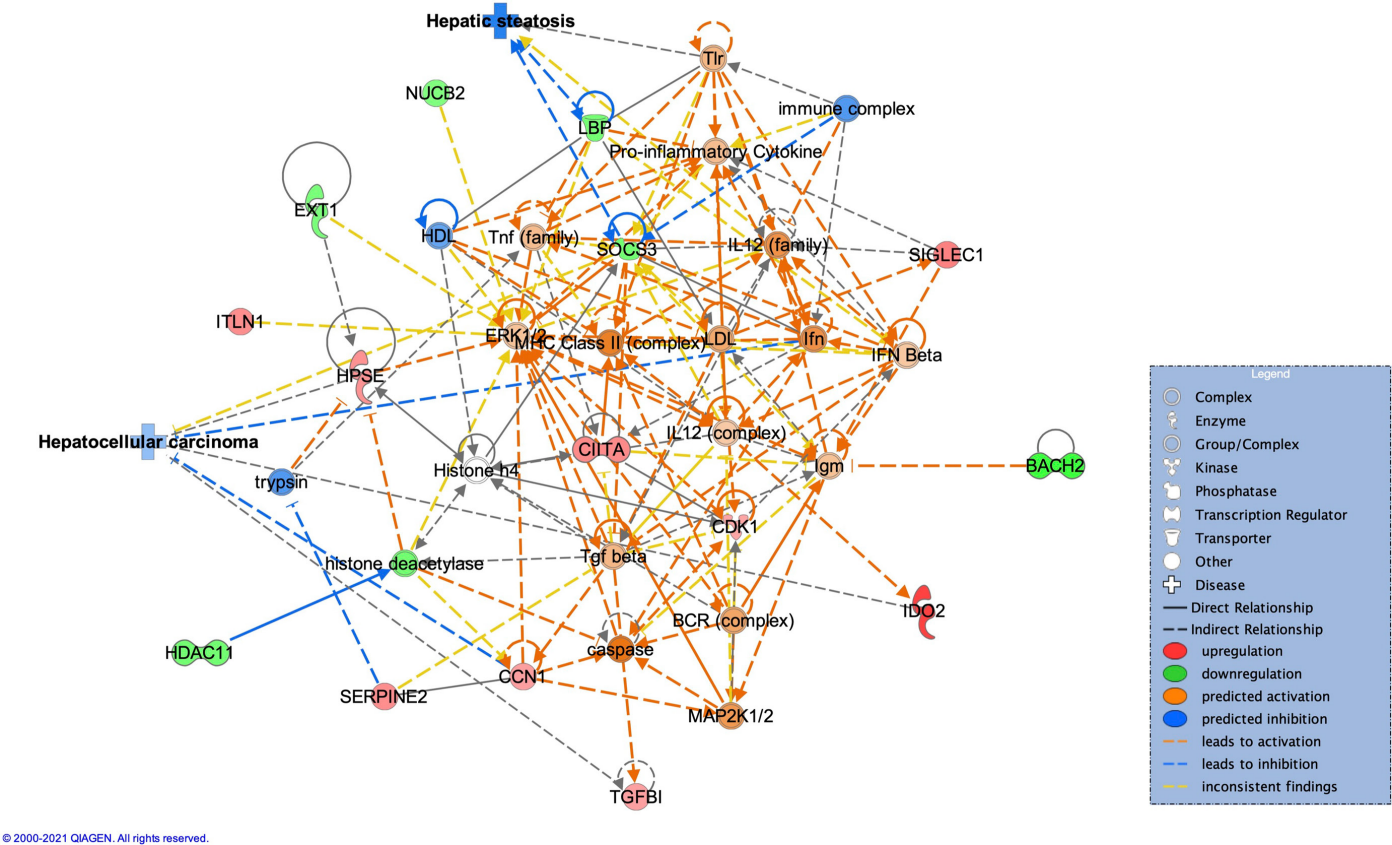
Achieving tolerance from sirolimus is predicted to decrease HCC (*p* = 1.30e‐02) and hepatic steatosis (*p* = 5.60e‐02) in the liver, based on the overlay with the disease and function database. Comparison of tolerance versus sirolimus timepoint. Network 2: Cellular Growth and Proliferation, Connective Tissue Development and Function, Tissue Development

**FIGURE 4:**
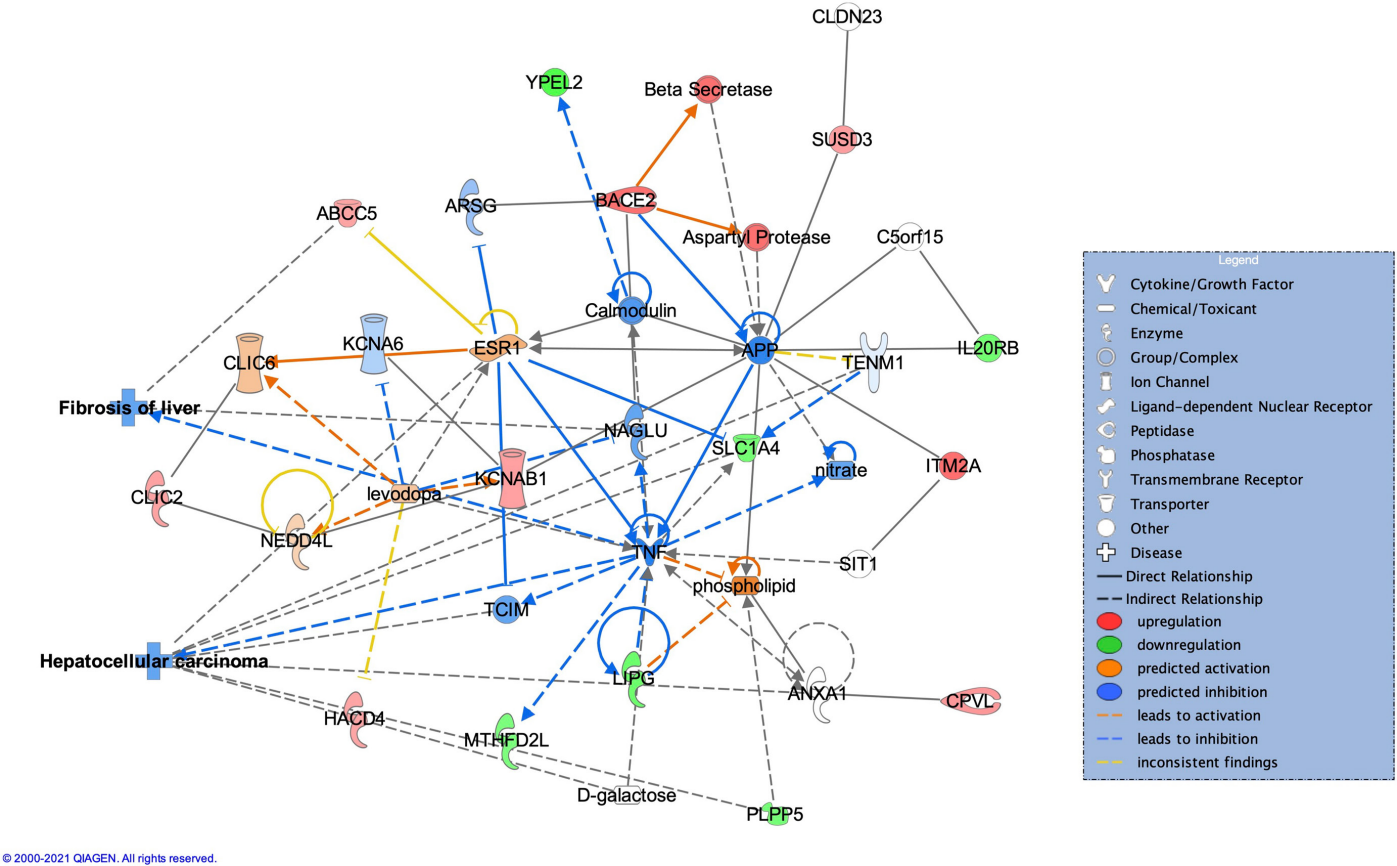
Achieving tolerance from sirolimus is predicted to decrease HCC (*p* = 9.83e‐03) and fibrosis of liver (*p* = 2.91e‐02), based on the overlay with the disease and function database. Comparison of tolerance versus sirolimus timepoint. Network 3: Developmental Disorder, Hereditary Disorder, Metabolic Disease

When comparing these two groups in liver tissue, 77 DEGs were identified (48 upregulated, 29 downregulated in tolerance group). Tolerance was found to be associated with cancer (*p*‐value range = 6.56e‐03‐1.51e‐09), organismal injury and abnormalities (*p*‐value range = 6.56e‐03‐2.14e‐07). Network analysis identified six networks associated with DEGs (Appendix S1). Of these, Network 2 (score = 31) was associated with Cellular Growth and Proliferation, Connective Tissue Development and Function, Tissue Development, with 9 upregulated and 6 downregulated genes (Figure 3). Network overlay with diseases and functions predicted a decrease in HCC (p‐value = 1.30e‐02) and hepatic steatosis (*p*‐value = 5.60e‐02) acquired by tolerance, with the DEGs involved in cancer and HCC in particular, or in hepatic steatosis being shown in Appendix S1. Based on the links between the molecules in the network and these diseases, inactivation of HCC is due to overexpressed CCN1/CYR61 (FC = 1.55), cellular communication network factor 1, a tumor suppressor in HCC.^12^ Upregulation of CCN1/CYR61 was associated with suppression of hepatocarcinogenesis, by limiting the proliferation of oncogenic hepatocytes.^13^ Potential decrease in HCC is also linked to prediction in activation of interferon (Ifn), based on the previous studies that found interferon to act as suppressor of carcinogenesis in HCV‐related HCC patients.^14^ In this network, the decrease in steatosis is linked to downregulated SOCS3 (FC = −1.49), suppressor of cytokine signaling 3, shown to play a role in limiting liver steatosis by inhibition of STAT3 activation,^15^ and to downregulated LBP (FC = −1.47), lipopolysaccharide binding protein, with higher levels of LBP being linked to increased liver steatosis in NAFLD patients.^16^ Showing a similar effect, network 3 (score = 28) associated with Developmental Disorder, Hereditary Disorder, Metabolic disease, with 8 upregulated and 6 downregulated genes, reflected a decrease in HCC (*p*‐value = 9.83e‐02) and fibrosis of liver (*p*‐value = 2.91e‐02) in tolerance group (Figure 4), due to predicted inactivation of tumor necrosis factor, TNF, previously shown to affect HCC development and recurrence.^17^

When performing tolerance versus sirolimus comparison analysis in blood samples, the cancer, organismal injury and abnormalities, were also top diseases and disorders found significantly associated with tolerance group (*p*‐value range = 2.63e—02‐2.39e‐06). In terms of networks determined by the DEGs between the two groups, the one involved similarly as in liver, in Cellular growth and Proliferation, Tissue Morphology, Organismal Functions was overlapped with diseases and functions from IPA database. Neoplasia of hepatocytes was predicted to be significantly inhibited (*p* = 5.61e‐03) (Figure S2) and linked in the network to transcription regulator HNF4A, hepatocyte nuclear factor 4 alpha, known to suppress the development of HCC.^18^ HNF4A was not among the DEGs from our analysis but rather was predicted to be activated by IPA. Liver tumor and fibrosis of liver were also linked to HN4A and inhibited in blood, but not at significant level.

## 4 DISCUSSION

In this study, we use a network analysis approach to examine longitudinal changes in hepatic gene expression during a protocol that resulted in ultimate weaning off of immunosuppression. This longitudinal analysis provides a natural setting to determine molecular profiles that portend cancer risk. Our study reveals molecular evidence of more favorable malignancy risk profiles as LT recipients transition from CNIs to mTOR‐I and tolerance. More specifically, our network analysis reveals favorable changes in hepatic DNA replication and repair genes and generally in HCC upon achieving tolerance. Whether conversion to sirolimus or even full withdrawal has a clinical benefit on de novo malignancy rates or other complications is yet to be determined. While our study provides biological rationale, clinical benefit of mTOR‐I conversion with or without withdrawal needs to be tested in prospective studies.

Our findings on cell cycle and DNA repair gene expression profiles improving with transition to tolerance are concordant with previous literature confirming that genetic variants in the cell cycle confer susceptibility to cancer (lung, ovarian, etc.).^19^, ^20^, ^21^ The cancer‐promoting effect of CNIs, previously described, and may be proportional to trough levels.^22^ Various mechanisms have been proposed, including inhibition of the effector immune responses,^23^, ^24^, ^25^ upregulation of transforming growth factor‐β (TGF‐B),^26^ downregulation of CXCR3‐B,^27^, ^28^ and overexpression of vascular endothelial growth factor (VEGF) that promotes angiogenesis.^29^ Contrary to tacrolimus, sirolimus forms a complex FKBP‐12 protein^30^ that can bind the kinase mTOR blocking the downstream signaling pathway, crucial for carcinogenic processes such as cell growth and proliferation, cellular metabolism and angiogenesis.^31^ Due to its antiproliferative effects,^12^ the putative advantages of using sirolimus as an alternative immunosuppressant to decrease the risk of malignancy have been previously described.^32^, ^33^, ^34^, ^35^ The inhibition of mTOR may have antiproliferative effects with several mechanisms including a selective decrease in the translation of mRNAs (such as c‐myc, VEGF) essential to tumorigenesis and a decreased phosphorylation of cyclin D1 leading to cycle progression arrest in cancer cells.^36^ In fact, conversion of tacrolimus to sirolimus has been shown previously by our group to result in decreased pro‐cancer gene expression, including genes such as eIF2 as well as genes along the mTOR pathway which become downregulated.^37^

This exploratory study provides mechanistic and biological data but is limited by the small number of highly select LT recipients studied. This precluded the ability to correlate molecular cancer risk with actual clinical differences in cancer rates between the cohorts, or metabolic risk with cardiovascular events. Longer term follow‐up with larger cohorts is needed to correlate biological and clinical data. Another limitation is that we have inferred changes in cancer‐associated genes in liver in the same individual under different immune conditions to be reflective of systemic effects on cancer susceptibility. Nonetheless, gene expression data from both the liver and PBMCs in the same individual was followed longitudinally, offering a unique opportunity to examine how differences in immunosuppression affect gene expression patterns.

The key finding is that patients have favorable changes in DNA repair, replication‐associated genes upon achieving tolerance, which we have uncovered using a network analysis approach on transcriptomic data from the liver. We thereby use this clinical setting to show how immunosuppression affects cancer risk molecular profiles in a single individual longitudinally. Future validation will include assessment as to whether these gene expression patterns correlate with reduced malignancy risk in patients who are successfully converted off CNI therapy and ultimately achieve tolerance.

## AUTHOR CONTRIBUTIONS

MB, EP, JL study design and writing of manuscript; EP, PP, JY, CB and SK data collection, analysis and compiling; MB, EP, CB and JL input into study design and final manuscript.

## CONFLICT OF INTEREST

Authors declare no conflict of interest.

## ETHICS STATEMENT

A written informed consent was obtained from patients for use of liver biopsy tissue and peripheral blood samples. The study protocol was approved by the Northwestern Institutional Review Board and was performed in accordance with the Declaration of Helsinki (https://clinicaltrials.gov/ct2/show/NCT02062944).

## Supporting information


Figure S1

Figure S2

Appendix S1

## Data Availability

Raw microarray data will be available upon request.
