# Supplementary figures and images for: Achieving tolerance modifies cancer susceptibility profiles in liver transplant recipients
Source: Cancer Med. 2022 Oct 7;12(4):5150–7. doi: 10.1002/cam4.5271 (PMC9972022; doi:10.1002/cam4.5271)

Supplementary Figure 1

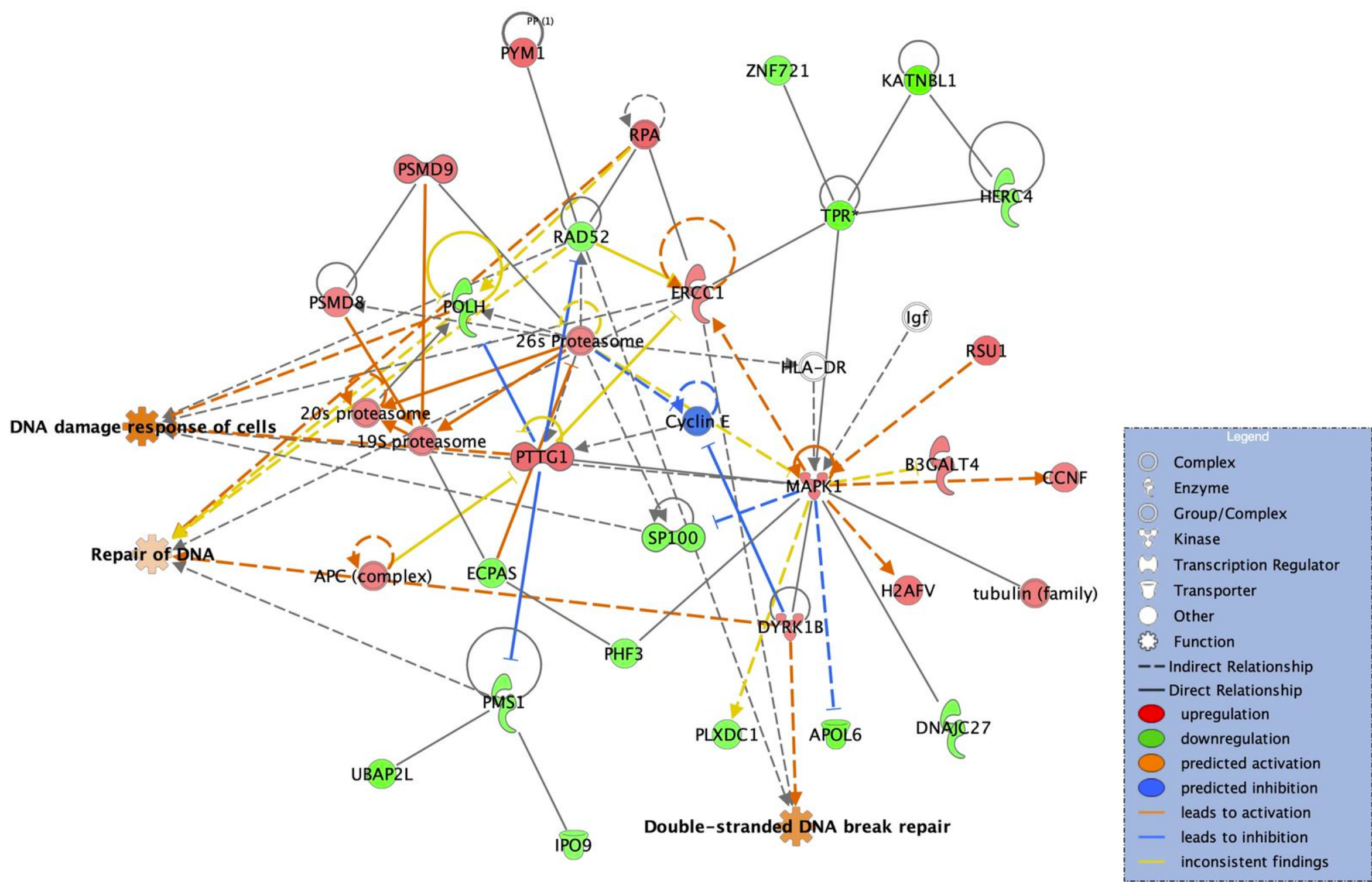

Supplement: Supplementary file 1 — Figure S1 [file CAM4-12-5150-s001.pdf]

Supplementary Figure 2

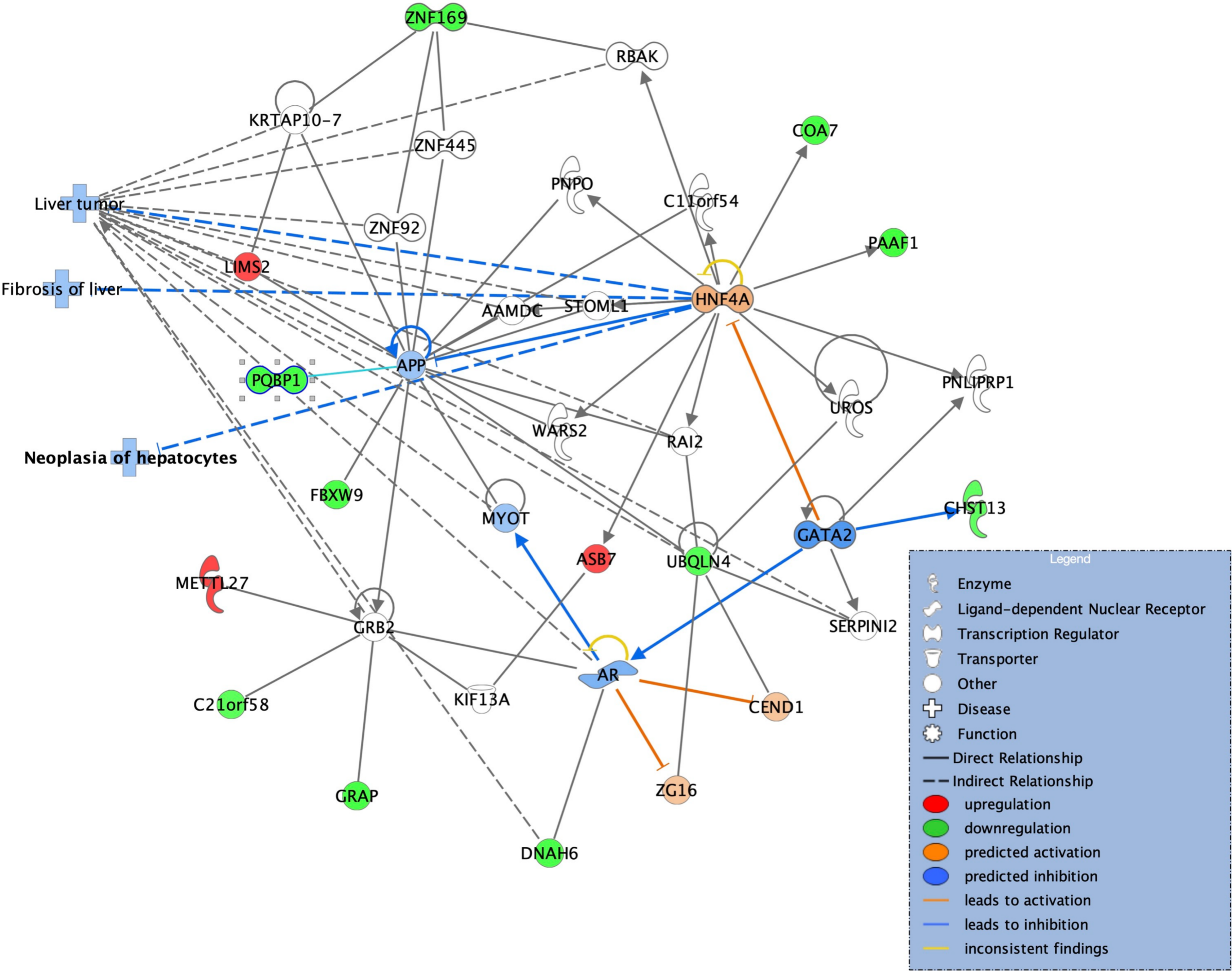

Supplement: Supplementary file 2 — Figure S2 [file CAM4-12-5150-s002.pdf]
