# Supplementary material for: Achieving tolerance modifies cancer susceptibility profiles in liver transplant recipients
Source: Cancer Med. 2022 Oct 7;12(4):5150–7. doi: 10.1002/cam4.5271 (PMC9972022; doi:10.1002/cam4.5271)
Supplement: Supplementary file 3 — Appendix S1 [file CAM4-12-5150-s003.docx]

**Achieving Tolerance Modifies Cancer Susceptibility Profiles in Liver Transplant Recipients**

**Mamatha Bhat^1^, Elisa Pasini^1^, Preya Patel^1^, Jeffrey Yu^1^, Cristina Baciu^1^, Sunil M. Kurian^3^, Josh Levitsky^4,5^**

^1^Multi Organ Transplant Program and Division of Gastroenterology, University Health Network and University of Toronto, Toronto, Canada

^2^Scripps Clinic Bio-Repository & Transplantation Research, Scripps Clinic & Green Hospital, La Jolla, CA, USA

^3^Division of Gastroenterology & Hepatology, Department of Medicine, Northwestern University Feinberg School of Medicine, Chicago, Illinois

^4^Comprehensive Transplant Center, Department of Surgery, Northwestern University Feinberg School of Medicine, Chicago, Illinois

**Corresponding Author**

Josh Levitsky, MD

Comprehensive Transplant Center

Division of Gastroenterology & Hepatology

Northwestern University Feinberg School of Medicine

Chicago, Illinois

Josh.Levitsky@nm.org

**Supporting Information**

**Supplementary Table 1.** Pro- and anti-cancer differentially expressed genes (Tolerance versus Tacrolimus) involved in Cell Cycle, DNA Replication and Repair network.

| Gene | Fold  Change | Modulation | Pro-cancer | Anti-cancer | PMID - cancer | PMID - HCC |
| --- | --- | --- | --- | --- | --- | --- |
| BLM | 1.304 | up | Y |  | | 32565735 |
| CDK9 | -1.199 | down | Y |  |  | 32048975 28940993 |
| DLGAP5 | 1.222 | up | Y |  |  | 24324629 |
| HOXC9 | 1.119 | up | Y |  | 31414766  27162534  32816159 | |
| Hsp70 |  | predicted activation | Y |  |  | 25798051 |
| KIF14 | -1.275 | down | Y |  |  | 23414349 |
| LINC00475 | -1.187 | down | Y |  | 32849915 |  |
| PAX9 | -1.204 | down | Y | Y | 29055049  18979497 | |
| TNRC6A | 1.175 | up | Y | Y | 31510013  29907771 |  |
| ZNF384 | -1.156 | down | Y |  |  | 31168049 |
| Ck2 | 1.208 | up |  | Y | 31684170 |  |
| L3MBTL1 | -1.231 | down |  | Y | 21837478 29737552 |  |
| MAP1S | -1.301 | down |  | Y |  | 28386016 |
| MSX1 | -1.231 | down |  | Y | 30127625 |  |
| PPP1R7 | -1.117 | down |  | Y | 19857786 30500680 |  |
| RB1 | 1.141 | up |  | Y |  | 24838394 |
| Rnr |  | predicted inactivation |  | Y |  | 28878246 |
| TNRC6A | 1.175 | up | Y | Y | 31510013 29907771 |  |

**Supplementary Table 2**. Differentially expressed genes (Tolerance versus Tacrolimus) involved in Neoplasia of cells.

| Gene | Fold  Change | Modulation | Pro-cancer | Anti-cancer | PMID - cancer | PMID - HCC |
| --- | --- | --- | --- | --- | --- | --- |
| BLM | 1.304 | up | Y |  | | 32565735 |
| SMURF2 | 1.304 | up |  | Y | 29758012 31409643 | 23781096 |
| SSBP1 | 1.288 | up | Y | Y | 26676758 | 21242961 |
| ETV6 | 1.253 | up | Y |  | 32326970 | |
| let-7 | 1.236 | up | Y |  |  | 32917856 29466730 |
| RB1 | 1.141 | up |  | Y |  | 23414349 |
| NR0B1 | -1.124 | down | Y |  | 19644015 |  |
| PAX9 | -1.204 | down |  |  | 29055049  18979497 | |
| ZNF503 | -1.291 | down | Y |  | 31510013  29907771 | 31312355 |
| STAB2 | -1.488 | down | Y |  |  | 23870052 |

**Supplementary Table 3.** Top 10 networks associated with differentially expressed genes (Tolerance vs Tacrolimus) in blood.

| Network # | Top Diseases and Functions | Score | Focus Molecules |
| --- | --- | --- | --- |
| 1 | Cardiovascular Disease, Organismal Injury and Abnormalities, Connective Tissue Disorders | 58 | 35 |
| 2 | Cellular Assembly and Organization, Skeletal and Muscular System Development and Function, Developmental Disorder | 45 | 30 |
| 3 | Developmental Disorder, Hereditary Disorder, Neurological Disease | 42 | 29 |
| 4 | Cellular Assembly and Organization, Cellular Function and Maintenance, Cellular Development | 42 | 29 |
| 5 | Metabolic Disease, Organismal Injury and Abnormalities, Developmental Disorder | 38 | 27 |
| 6 | RNA Post-Transcriptional Modification, Gene Expression, Connective Tissue Disorders | 38 | 27 |
| 7 | Energy Production, Nucleic Acid Metabolism, Small Molecule Biochemistry | 38 | 27 |
| 8 | DNA Replication, Recombination, and Repair, Cell Morphology, Cellular Assembly and Organization | 36 | 26 |
| 9 | Post-Translational Modification, Protein Degradation, Protein Synthesis | 36 | 26 |
| 10 | Cancer, Gastrointestinal Disease, Hereditary Disorder | 36 | 26 |

**Supplementary Table 4**. Differentially expressed genes (Tolerance vs Sirolimus in liver tissue) in Network#2 (Figure 2) associated with HCC or Liver steatosis. In red are shown the upregulated genes, in green – the downregulated genes, in orange -predicted activation.

| **Category** | **Gene** | **PMID** | **HCC** | **Cancer** | **Steatosis** |
| --- | --- | --- | --- | --- | --- |
| **HCC** | CCN1 | 26028023 | Yes |  |  |
|  |  | 29286082 | Yes |  |  |
|  |  | 22540002 | Yes |  |  |
|  | Ifn | 31289685 | Yes | HCV-related HCC | |
|  |  | 25963067 | Yes | HCV-related HCC | |
|  | IDO2 | 30266763 |  | Yes |  |
|  |  | 32468023 |  | Yes |  |
|  | HPSE | 32895190 | Yes |  |  |
|  |  | 29158804 | Yes |  |  |
|  |  | 25149140 | Yes |  |  |
| **Steatosis** | SOCS3 | 29550470 |  |  | Yes |
|  |  | 32272865 |  |  | Yes |
|  | LBP | 27404046 |  |  | Yes |
|  |  | 30235828 |  |  | Yes |
|  |  | 30896956 |  |  | Yes |
|  | IL12 | 20034047 |  |  | Yes |
|  | Tlr | 32839596 |  |  | Yes |
|  |  | 27890642 |  |  | Yes |

**Supplementary Table 5**. Networks associated with differentially expressed genes (tolerance vs sirolimus) in liver tissue.

| Network # | Top Diseases and Functions | Score | Focus Molecules |
| --- | --- | --- | --- |
| 1 | Cellular Movement, Hematological System Development and Function, Immune Cell Trafficking | 36 | 17 |
| 2 | Cellular Growth and Proliferation, Connective Tissue Development and Function, Tissue Development | 31 | 15 |
| 3 | Developmental Disorder, Hereditary Disorder, Metabolic Disease | 28 | 14 |
| 4 | Cell Morphology, Connective Tissue Development and Function, Skeletal and Muscular System Development and Function | 21 | 11 |
| 5 | Embryonic Development, Organismal Development, Endocrine System Development and Function | 18 | 10 |
| 6 | Connective Tissue Disorders, Inflammatory Disease, Inflammatory Response | 16 | 9 |

**Supplementary figure legends**

**Supplementary Figure 1:** Tolerance is predicted to improve DNA damage response of cells (p=2.92e-06), Repair of DNA (p=4.20e-04), Double-stranded DNA break repair (p=2.93e-03) in blood (tolerance versus tacrolimus timepoint). Network 8 in IPA: DNA Replication, Recombination, and Repair, Cell Morphology, Cellular Assembly and Organization

**Supplementary Figure 2:** Achieving tolerance from sirolimus (tolerance versus sirolimus group comparison) is predicted to significantly decrease neoplasia of hepatocytes (p=5.61e-03) in blood. Network 7: Cellular Growth and Proliferation, Tissue Morphology, Organismal Functions.
